# Tumours with loss of MSH6 expression are MSI-H when screened with a pentaplex of five mononucleotide repeats

**DOI:** 10.1038/sj.bjc.6605988

**Published:** 2010-11-16

**Authors:** J-F You, O Buhard, M J L Ligtenberg, C M Kets, R C Niessen, R M W Hofstra, A Wagner, W N M Dinjens, C Colas, O Lascols, A Collura, J-F Flejou, A Duval, R Hamelin

**Affiliations:** 1 INSERM, UMRS 938-Centre de Recherche Saint-Antoine, Equipe ‘Instabilité des Microsatellites et Cancers’, 184 rue du Faubourg Saint-Antoine, Paris F-75012, France; 2 Université Pierre et Marie Curie-Paris6, Paris, France; 3 Colorectal Section, Department of Surgery, Chang Gung Memorial Hospital at Linkou, Chang Gung University College of Medicine, Taoyuan, Taiwan; 4 Department of Human Genetics, Radboud University Nijmegen Medical Centre, Nijmegen, The Netherlands; 5 Department of Genetics, University Medical Center Groningen, University of Groningen, Groningen, The Netherlands; 6 Department of Clinical Genetics, Erasmus MC, University Medical Center, Rotterdam, The Netherlands; 7 Department of Pathology, Josephine Nefkens Institute, Erasmus MC, University Medical Center, Rotterdam, The Netherlands; 8 AP-HP, Hôpital Pitié-Salpêtrière, Service d'Oncogénétique, Paris, France; 9 AP-HP, Hôpital Saint-Antoine, Service de Biochimie, Paris, France; 10 AP-HP, Hôpital Saint-Antoine, Service d'Anatomie et Cytologie Pathologiques, Paris, France

**Keywords:** microsatellite instability, Lynch syndrome, MSH6

## Abstract

**Background:**

Microsatellite instability (MSI) is commonly screened using a panel of two mononucleotide and three dinucleotide repeats as recommended by a consensus meeting on MSI tumours held at the National Cancer Institute (Bethesda, MD, USA). According to these recommendations, tumours are classified as MSI-H when at least two of the five microsatellite markers show instability, MSI-L when only one marker shows instability and MSS when none of the markers show instability. Almost all MSI-H tumours are characterised by alterations in one of the four major proteins of the mismatch repair (MMR) system (MLH1, MSH2, MSH6 or PMS2) that renders them MMR deficient, whereas MSI-L and MSS tumours are generally MMR proficient. However, tumours from patients with a pathogenic germline mutation in *MSH6* can sometimes present an MSI-L phenotype with the NCI panel. The MSH6 protein is not involved in the repair of mismatches of two nucleotides in length and consequently the three dinucleotide repeats of the NCI panel often show stability in MSH6-deficient tumours.

**Methods:**

A pentaplex panel comprising five mononucleotide repeats has been recommended as an alternative to the NCI panel to determine tumour MSI status. Several studies have confirmed the sensitivity, specificity and ease of use of the pentaplex panel; however, its sensitivity for the detection of MSH6-deficient tumours is so far unknown. Here, we used the pentaplex panel to evaluate MSI status in 29 tumours known to harbour an MSH6 defect.

**Results:**

MSI-H status was confirmed in 15 out of 15 (100%) cases where matching normal DNA was available and in 28 out of 29 (97%) cases where matching DNA was not available or was not analysed.

**Conclusion:**

These results show that the pentaplex assay efficiently discriminates the MSI status of tumours with an MSH6 defect.

Human tumours with a deficient DNA mismatch repair (MMR) system represent a subset of tumours with distinctive features at both the molecular and clinical levels. They are relatively frequent and account for 10–15% of common cancer types such as those arising in the colon, stomach and endometrium (Boland *et al*, 1998). The result of defective DNA MMR is the occurrence of frequent insertion–deletion mutations, especially in short repetitive DNA sequences called microsatellites. Tumours showing such mutations are therefore referred to as having microsatellite instability (MSI) (Boland *et al*, 1998). At the clinical level, MSI tumours are known to have better prognosis than non-MSI (or MSS for microsatellite stable) tumours (Popat *et al*, 2005) and may respond differently to adjuvant chemotherapy (Ribic *et al*, 2003). In most sporadic MSI tumours, the MMR defect arises because of the failure to express the *MLH1* gene due to somatic hypermethylation of its promoter (Kane *et al*, 1997). MSI tumours also arise in families with a genetic predisposition known as Lynch syndrome (Lynch and Lynch, 2004). Predisposed members of these families contain a germline mutation in one of the MMR genes, usually *MSH2* or *MLH1* and less frequently *MSH6* or *PMS2* (Peltomäki, 2005). Screening to determine defective MMR status is becoming increasingly common, especially with a view to identify families with Lynch syndrome. Two different strategies are used for this purpose. Immunohistochemistry (IHC) is used to detect the lack of expression of one or more MMR proteins, whereas MSI analysis is used to detect instability in microsatellite repeats. Pros and cons are inherent to either strategy (Shia, 2008; Zhang, 2008) and will not be discussed here, other than to state that these methods provide complementary information.

Following an NCI consensus meeting on MSI tumours, a panel of five microsatellites (two mononucleotide repeats and three dinucleotide repeats) was proposed to standardise the classification of MSI status in tumours (Boland *et al*, 1998). By comparing amplified tumour DNA profiles with matching normal DNA, a tumour is classified as MSI-H when it shows instability in at least two of the five markers and MSI-L or MSS when it shows instability at one or none of the markers, respectively (Boland *et al*, 1998). MSI-L tumours usually show instability at only one of the three dinucleotide markers and their morphological and clinical phenotype is indistinguishable from MSS tumours (Laiho *et al*, 2002). However, tumours that have a deficiency in MSH6 often do not show instability in dinucleotide repeats and therefore risk being classified as MSI-L or even MSS when analysed with the NCI panel containing only two potentially unstable mononucleotide repeats (Verma *et al*, 1999; Wu *et al*, 1999).

At a second consensus meeting, mononucleotide repeats were recognised as being more specific and sensitive for the determination of MSI status than dinucleotide repeats (Umar *et al*, 2004). A panel of five mononucleotide repeats was proposed and the conditions for their amplification in a single pentaplex PCR were described (Suraweera *et al*, 2002). However, the efficiency of the pentaplex panel for the detection of MMR-deficient tumours with an *MSH6* mutation is currently unknown and is the subject of the present investigation. Germline mutations in *MSH6* are relatively rare and estimated to account for 5–10% of Lynch syndrome cases. These in turn represent 2–7% of all colorectal cancers, indicating that germline mutations in *MSH6* account for ∼0.3% of cases. In the present study, 29 tumour samples with MSH6 deficiency were collected from three Dutch and one French cancer centres and evaluated for MSI status using the pentaplex panel.

## Materials and methods

### Tissue specimens

Formalin-fixed, paraffin-embedded tumour and matching normal (when available) samples were obtained from three Dutch (Radboud University Nijmegen Medical Centre, Nijmegen, the Netherlands; University Medical Center Groningen, Groningen, the Netherlands; Josephine Nefkens Institute, Erasmus MC, Rotterdam, the Netherlands) and one French cancer centre (Hôpital Saint-Antoine, Paris, France). All patients gave informed consent for the analysis. MMR-deficient protein(s) were determined by IHC analysis in all cases. As it is well established that MSI tumours deficient in MSH2 also show loss of MSH6 expression, the current study investigated only tumours that showed loss of MSH6 expression alone. A total of 29 tumours arising from different sites (colon, endometrium and urothelium) were selected, of which matching normal DNA was available for 15 cases. The fixation procedure, length of storage and DNA extraction method differed considerably between samples due to their different origin. Germline *MSH6* mutations were determined in most cases from peripheral blood samples using standard sequencing methods. DNA from 10 MSS tumours, and their matching normal DNA, was also analysed.

### Microsatellite analysis

Two versions of the pentaplex assay were used here to analyse the Dutch samples as previously described. The older version comprises the NR-21, BAT-26, BAT-25, NR-24 and NR-22 mononucleotide repeats with average PCR product sizes in Caucasian individuals of 103, 120, 124, 132 and 142 bp, respectively (Suraweera *et al*, 2002). The newer version of the assay comprises the NR-27, NR-21, NR-24, BAT-25 and BAT-26 repeats with average PCR product sizes in Caucasians of 87, 107, 126, 148 and 179 bp, respectively (Buhard *et al*, 2004, 2006). The new conditions were designed to avoid interference between different dyes during the laser scanning, and this assay is generally more convenient for use with high quality DNA extracted from frozen tissue samples. However, the larger PCR products of the new assay compared to the older pentaplex assay makes it more susceptible to DNA quality. Samples from the Saint-Antoine hospital were analysed using a commercial MSI detection kit (Promega France, Charbonnières-les-Bains, France), derived from the pentaplex assay and composed of NR-21, BAT-26, BAT-25, NR-24 and Mono-27. The latter mononucleotide repeat (localised within the *MAP4K3* gene) is different to NR-27 (localised within the 5′ UTR of the *inhibitor of apoptosis protein 1* gene). Tumours were defined as MSI when three or more of the five markers showed instability, regardless the pentaplex version that was used (Suraweera *et al*, 2002; Buhard *et al*, 2006). In the absence of comparison with matching normal DNA, these very stringent conditions avoid the occurrence of rare false-positive cases due to some ethnic variants.

## Results

### Pentaplex amplification efficiency depends on PCR product size

**Figure 1:**
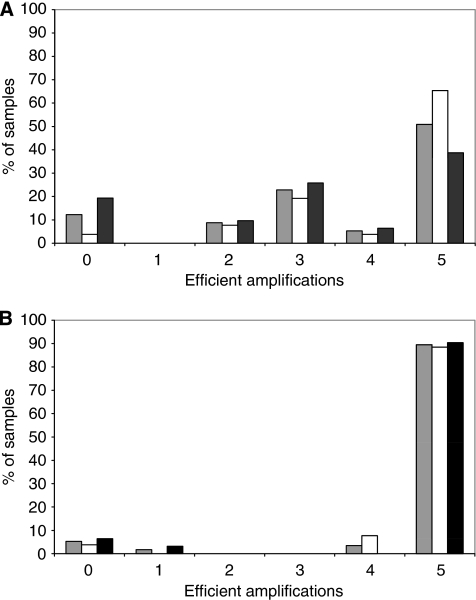
Comparative analysis of the amplification efficiency with old and new versions of the pentaplex assay. DNA extracted from 31 tumours (21 with an MSH6 defect and 10 classified as MSS) and from 26 matching normal tissues (Nijmegen and Groningen series) were analysed with new (**A**) or old (**B**) versions of the pentaplex assay. In each case, the percentage of samples that showed successful amplification of 0–5 of the markers is indicated: white columns, normal DNA; black columns, tumour DNA; grey columns, all samples. Three tumours and one normal DNA showed amplification of none or only one of the markers with both the old and new pentaplex assay versions and were excluded from further analysis. DNA samples that successfully amplified at four or five markers using the old assay were kept for analysis even if the amplification was not successful with the new assay version.

We first analysed samples from Nijmegen and Groningen (tumour DNA together with matching normal controls when available), with the new version of the pentaplex assay in which the size of the normal PCR products ranged from 87 to 179 bp (Buhard *et al*, 2006). Successful amplification of all five markers was achieved in only 51% of samples (Figure 1A). Not surprisingly, successful amplification was more commonly observed for the markers associated with shorter PCR products. The success of PCR amplification is highly dependent on sample fixation, length of storage and method of DNA extraction. As these conditions were quite variable for the different samples, it was decided to use the older pentaplex assay version in which the PCR product sizes are smaller (range 103–142 bp) (Suraweera *et al*, 2002). With this assay, successful amplification of the five markers was obtained in 90% of cases regardless of their origin from tumour or normal matching DNA (Figure 1B). DNA samples from the third Dutch series (Rotterdam) were therefore amplified directly using the older pentaplex assay, and all results described below on the Dutch samples were obtained using this assay.

### PCR profiles of normal DNA

**Figure 2:**
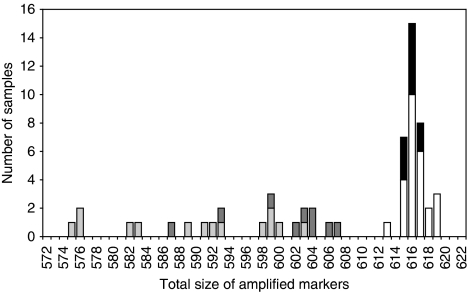
Total size of amplified markers using the old pentaplex assay. The sum of the sizes of the five PCR products obtained with the old pentaplex assay is indicated for normal DNA (white columns), DNA extracted from MSS tumours (black columns) or DNA extracted from MSH6-defective tumours (grey columns). In the latter case, light grey corresponds to tumours obtained from colon, and dark grey to tumours from the endometrium or urothelium.

The size of all PCR products was determined individually using Genescan software and was rounded up or down to the next integer. Amplification for all five markers was achieved in 23 out of 25 (92%) of the normal tissue samples. The size variation observed was 103–105 bp (NR-21), 116–118 bp (BAT-26), 122–124 bp (BAT-25), 130–131 bp (NR-24) and 141–142 bp (NR-22). These values define the quasi-monomorphic variation range (QMVR) for each marker in the germline DNA analysed in this study. They are slightly different from the values reported in our original manuscript. We have already shown that this is due to the use of different instrumentation and reagents (Buhard *et al*, 2006). These factors were not identical 8 years apart. The total variation range (TVR) as defined by the sum of the sizes of the five markers was 613–619 bp for normal DNA in the present analysis (Figure 2). None of the markers in any of the individuals in this study showed an allelic variant that was clearly outside of the QMVR. This has been found on rare occasions in the worldwide populations (Buhard *et al*, 2006). Although the ethnic origin of the Dutch patients analysed here was unknown, the majority were likely to be Caucasian. If present in a series, variant alleles should not be included for QMVR and TVR calculation in normal DNA.

### PCR profiles of DNA from MSS tumours

Amplification of the five markers was achieved in all 10 MSS tumour samples and matching normal DNA. The size of PCR products obtained with tumour DNA showed no size variation compared to those obtained from matching normal DNA. Without referring to matching normal DNA, each marker from each tumour was then assessed in terms of whether it was inside or outside the normal QMVR. The sum of the size of each amplification product was also compared to the TVR from normal DNA. Markers were always within the QMVR and the total size of the five amplified products varied from 615 to 617 bp. These were all within the normal TVR of 613–619 bp as defined above (Figure 2). As expected, MSS tumours could thus be classified without the need to refer to profiles from matching normal DNA. These results confirm those previously obtained with the original version of the pentaplex assay on 62 MSS gastrointestinal primary tumours and cell lines (Suraweera *et al*, 2002).

### PCR profiles of DNA from tumours with MSH6 deficiency

Of the 23 MSH6-defective tumour samples of Dutch origin, matching normal DNA was available for 15 cases. Successful amplification of the five markers was achieved for both the tumour and normal DNA in all 15 cases. Differences between tumour and matching normal DNA in terms of the size of PCR products for each marker are shown in Table 1A. Fourteen cases showed instability in all five markers, whereas the remaining case showed instability in four of the five markers. In some cases, the instability was only 1 bp in size. The non bold values in Table 1a indicate markers that were within the QMVR, as defined above for the normal DNA. These markers would have been recorded as stable in the absence of comparison with matching normal DNA. Bold values indicate markers that were outside the normal QMVR and would have been recorded as unstable, even in the absence of comparison with matching normal DNA. Thus, if analysis was performed without reference to matching normal DNA, instability at five or four markers was detected in 10 and five cases, respectively.

Normal matching DNA was not available or did not amplify correctly for eight additional tumours. We thus compared the size of the amplified products to the QMVR (Table 1B). Markers were considered unstable when they fell outside QMVR values (bold values) and stable when they were within QMVR values (non bold values). These eight MSH6-deficient tumours showed instability at five markers (three cases), four markers (four cases) and three markers (one case). As MSI was defined as three or more of the five markers showing instability, all eight tumours were considered to show MSI.

Using the original pentaplex assay, the MSH6-deficient samples thus displayed a total PCR product size of 575–607 bp (Figure 2, grey columns). This was clearly different to the total size observed for MSS tumours (Figure 2, black columns) and to the TVR of normal DNA (Figure 2, white columns; 613–619 bp). MSI tumours could be roughly divided into two groups showing total sizes of 575–593 bp and 598–607 bp. This subclassification showed some relation to tumour localisation (colon/small bowel *vs* endometrium/urothelium). Although not statistically significant (*P*=0.09, Fisher's exact test), this result confirms the already published observation that endometrial MSI tumours show a smaller amplitude of instability compared to colorectal MSI tumours (Wong *et al*, 2006).

Finally, we examined six cases from patients who underwent surgery for colorectal cancer at Saint-Antoine Hospital and whose tumours showed loss of MSH6 expression alone. These were evaluated for MSI using a commercial kit (Promega) that was based on the pentaplex assay (Table 1C). In the absence of normal DNA, the QMVR for the five markers in this kit was defined by the size of the PCR products in a series of about 1000 MSS tumours (as determined by IHC analysis), excluding obvious variant alleles due to ethnicity (not shown). The QMVR was 99–103 bp (NR-21), 114–116 bp (BAT-26), 121–126 bp (BAT-25), 130–133 bp (NR-24) and 150–154 bp (Mono-27), while the TVR was 614–632 bp. Three of the six MSH6-deficient samples had at least four of the markers whose size was outside the QMVR. These showed total values of 591, 597 and 604 bp. Two tumours had two markers outside the QMVR and showed total values of 610 and 613 bp, which were still lower than the normal TVR. Although not obviously MSI according to standard criteria, the results obtained with these two samples drew attention to the fact that further investigation was required to correctly define their MSI status. Although it showed a relatively low TVR (614 bp), the final sample did not present any characteristics to indicate it had MSI.

## Discussion

Determination of the MSI status of tumours is performed routinely in most cancer centres due to the clinical importance of this phenotype. For this purpose, IHC has an advantage over MSI screening in that it can indicate which MMR gene is altered. MSI analysis can also be more time- and labour-consuming than IHC. However, on some occasions, germline missense mutations in an MMR gene can lead to functional inactivation of the protein without affecting its stability and therefore its expression level. Our view is that IHC and MSI screening methods provide complementary information regarding defective MMR and should therefore be performed in parallel, although this is still a matter of ongoing debate.

The NCI panel of markers proposed at an international consensus meeting served as an important step in standardising the MSI determination process (Boland *et al*, 1998). Nevertheless, at a follow-up NCI workshop, it was widely acknowledged that dinucleotide microsatellites, of which there are three in the NCI panel, were less sensitive and specific than mononucleotide repeats for the determination of MSI status (Umar *et al*, 2004). One of the major objections levelled against the original NCI panel concerned the screening of tumours with defective *MSH6*, one of the four major MMR genes responsible for Lynch syndrome. The MSH6 protein participates in the correction of base–base mismatches and single nucleotide deletions/insertions, but not in the repair of larger deletion/insertion loops (Drummond *et al*, 1995). Instability involves one unit of repeated sequences and dinucleotide repeats are unstable in units of 2 bp. Hence, the corresponding mismatches are not recognised by the MutS*α* complex (MSH2/MSH6), but by the MutS*β* complex (MSH2/MSH3). Consequently, tumours with MSH6 deficiency are generally stable at dinucleotide repeats, and their MSI status is therefore debatable. There is clearly a need for further work to identify markers that are specific for MSH6-deficient tumours.

Our group recently proposed a panel of five mononucleotide repeats to determine MSI and has described the conditions necessary to amplify these markers in a single pentaplex PCR (Suraweera *et al*, 2002; Buhard *et al*, 2006). The sensitivity and specificity of this pentaplex assay, together with its ease of use, has led to its widespread adoption for the determination of MSI status in tumours (Murphy *et al*, 2006; Xicola *et al*, 2007; Goel *et al*, 2010). Preliminary data had suggested that MSH6-deficient tumours could be detected using mononucleotide repeats, but these studies were performed with a limited number of markers and tumours (Verma *et al*, 1999; Berends *et al*, 2002; Kets *et al*, 2006; Mead *et al*, 2007). The present study represents the first systematic analysis of a large series of MSH6-deficient tumours using the five mononucleotide repeats that comprise the pentaplex assay.

Our study was restricted to tumour samples that showed a deficiency in MSH6 expression only, as identified by IHC. These tumours are relatively rare and were obtained from four different cancer centres in the Netherlands and France. Due to their diverse origins, DNA extracted from these samples was not always of sufficient quality to allow amplification of relatively large PCR products. The original version of the pentaplex assay involving smaller PCR products was therefore used to evaluate MSI status. The five markers of the assay (or a commercial kit based on the assay) were amplified and their size was evaluated in 29 MSH6-defective tumour samples. The assay demonstrated MSI-H status in 15 out of 15 cases (100%), in which matching normal DNA was available, with instability in at least four of the five markers.

When the pentaplex assay conditions were originally described, these markers were shown to be quasi-monomorphic in normal DNA from most populations worldwide (Buhard *et al*, 2006). In the absence of matching normal DNA, MSI-H status was classified when tumours showed at least three of the five markers with sizes outside the QMVR deduced from a large series of unrelated normal DNA. These conditions are more stringent than those for the NCI panel of markers, where tumours are considered MSI-H if they show instability in at least two of the five markers (Boland *et al*, 1998). At the same time, they avoid the occurrence of rare false-positive cases due to possible ethnic variants. According to this rule, 26 out of 29 (89.5%) of the MSH6-defective samples evaluated in the present study were classified as MSI-H under conditions in which the matching normal DNA was not available or was not analysed. Two of the remaining cases showed instability at two markers and hence would not have been classified as MSI, although they would have attracted attention due to the larger than normal ‘stuttering’ observed in the three ‘stable’ markers. The final tumour showed no signs of instability in the five markers, probably because the tumour content of the sample was below 5–10%, the known limit of detection in these conditions (Brennetot *et al*, 2005). Unfortunately, additional material was not available for this patient.

The total size of the five PCR products obtained using the original pentaplex assay or commercial kit was calculated for each MSH6-deficient tumour in order to compare it with the TVR observed in normal DNA. In 28 out of 29 cases (96.5%), these totals were outside the normal TVR, indicating a positive MSI status for these samples.

It is generally considered that most, but not all tumours from Lynch family members are MSI-H. False-negative cases may be due to the use of NCI panel markers, particularly for MSH6-defective samples. The pentaplex assay used here is likely to contain the best set of markers described to date for the identification of MSI tumours, regardless of their MMR defect. Moreover, matching normal DNA is not mandatory in most cases. The superior performance of the pentaplex assay over the NCI panel is particularly evident for tumours with defective MSH6 and for endometrial cancer samples. For rare cases that display borderline instability and for which normal matching DNA is not available, the total size of PCR products for the five markers can be compared with the TVR obtained from unrelated normal DNA.

## Figures and Tables

**Table 1:** Sizes of PCR products obtained with the pentaplex assay in MSH6-defective tumours

**(A)**								
**ID DNA**		***MSH6* mutation**	**NR21**	**BAT26**	**BAT25**	**NR24**	**NR22**	**Total**
Y708-D4826^a^	Colon	c.2672delT,2674delT (p.Ile891fsX8)	**−9**	**−12**	**−8**	**−3**	**−8**	575
Y708-D4824^a^	Colon	c.2672delT,2674delT (p.Ile891fsX8)	**−9**	**−8**	**−8**	**−3**	**−7**	576
M03-121B^b^	Colon	ND	**−9**	**−3**	**−8**	**−7**	**−4**	576
K4-033T	Colon	c.3514dup (p.Arg1172fsX5)	**−9**	**−9**	**−8**	**−3**	**−5**	582
K120T	Colon	c.651dup (p.Lys218X)	**−7**	**−10**	**−4**	**−4**	**−3**	589
K97T	Colon	c.651dup (p.Lys218X)	**−4**	**−9**	**−4**	**−6**	**−3**	591
Y3039-D7713	Colon	c.1190_1191del (p.Tyr397CysfsX3)	**−4**	**−6**	**−5**	**−5**	−1	598
K5-034T	Colon	c.261−?_457+?dup (duplication exon 2)	**−7**	**−3**	0	**−4**	**−4**	599
K5-063T	Colon	c.3438+1G>A (splicing site mutation)	**−4**	**−6**	**−3**	**−2**	**−3**	599
K3-120T	Colon	c.3273dup (p.Lys1092X)	**−3**	**−4**	**−3**	**−3**	**−1**	600
K5-145T	Endometrium	c.3261del (p.Phe1088SerfsX2)	**−3**	**−3**	**−5**	**−2**	**−3**	602
K6-097T	Colon	c.4001G>A (p.Arg1334Gln splicing site mutation)	**−4**	**−4**	**−1**	**−4**	−1	603
Y37-D2057	Endometrium	c.651dup (p.Lys218X)	**−3**	**−2**	**−4**	**−2**	**−2**	603
K3-019T	Urothelium	c.1−?_457+?del (deletion exon 1 and 2)	**−4**	**−2**	−1	**−1**	**−4**	604
M03-121C^b^	Endometrium	ND	**−3**	**−5**	**−3**	**−1**	−1	604
								
**(B)**								
**ID DNA**		***MSH6* mutation**	**NR21**	**BAT26**	**BAT25**	**NR24**	**NR22**	**Total**
Y88-D3251	Colon	c.3263dupT (p.Glu1090ArgfsX3)	**99**	**108**	**118**	**120**	**138**	583
M04-293B	Endometrium	ND	**98**	**111**	**115**	**127**	**136**	587
K4-029T	Small bowel	c.2815C>T (p.Gln939X)	**99**	**111**	**119**	**127**	**136**	592
Y1-D3737^c^	Urothelium	c.3772C>T (p.Gln1258X)	**99**	**108**	**118**	131	**137**	593
Y725-D940	Colon	c.651dup (p.Lys218X)	**96**	**108**	**120**	131	**138**	593
M02-285B	Endometrium	c.1614_1616delTCinsAG (p.Tyr538X)	103	**112**	**118**	**127**	**139**	599
Y1-D3736^c^	Urothelium	c.3772C>T (p.Gln1258X)	104	**114**	**121**	**129**	**138**	606
M04-87T	Endometrium	c.1784del (p.Leu595TyrfsX15)	**102**	**114**	123	130	**138**	607
								
**(C)**								
**ID DNA**		***MSH6* mutation**	**NR21**	**BAT26**	**BAT25**	**NR24**	**Mono27**	**Total**
06R0110	Colon	c.560dup (p.Ile188AspfsX2)	**96**	**109**	**118**	**123**	**145**	591
07D280	Colon	ND	**94**	**110**	**120**	**126**	**147**	597
07G8474	Colon	ND	**95**	**110**	**120**	131	**148**	604
08G664	Colon	ND	99	**110**	**120**	130	151	610
07G8779	Colon	c.3264_3270del (p.Glu1090LysfsX23)	101	114	**120**	131	**147**	613
01G5462	Colon	ND	99	114	121	130	150	614

Abbreviation: ND=not done.

1a and 1b show Dutch tumour series analysed with the old pentaplex assay in comparison to matching normal DNA (1a) or to normal DNA QMVR (1b). 1c shows French tumour series analysed with the commercial MSI kit and compared to normal DNA QMVR. In each case, the total size of amplified markers is indicated and the tumours are classified according to the increasing size of these totals. Bold values indicate PCR products that were classified as showing instability, even if they were not compared to matching normal DNA (in 1a), as they were outside the normal QMVR. Non bold values indicate PCR products within the normal QMVR and classified as showing no instability when not compared to matching normal DNA.

^a,b,c^Different tumours from the same patient. Germline *MSH6* mutations are indicated in most cases.
